# When the Liver Flares: Inflammatory and Immunometabolic Mechanisms Driving the Transition from MASLD to MASH

**DOI:** 10.1007/s10753-026-02545-z

**Published:** 2026-06-16

**Authors:** Cristina Vecchio, Anteneh Nigussie Sheferaw, Emmanuel Kivumbi, Ian Stoppa, Deepika Pantham, Foteini Christaki, Alessia Provera, Umberto Dianzani, Salvatore Sutti

**Affiliations:** Dept. of Health Sciences, University of Piemonte Orientale, Novara, Italy

**Keywords:** MASLD, MASH, NAFLD, Inflammation, Immunity, Hepatokines, Immunometabolism

## Abstract

The prevalence of metabolic dysfunction-associated steatotic liver disease (MASLD) is steadily increasing worldwide, primarily due to the ongoing obesity pandemic. Although MASLD may initially present as a relatively benign condition, it has the potential to progress to metabolic dysfunction-associated steatohepatitis (MASH), a more severe form that can lead to cirrhosis and hepatocellular carcinoma (HCC). A variety of factors contribute to the pathogenesis of MASLD, including gut dysbiosis, insulin resistance, dyslipidemia, lipotoxicity, and oxidative stress. Among these, recent evidence highlights chronic inflammation as a key driver of disease progression toward advanced stages. Global scientific efforts have begun to uncover the molecular mechanisms sustaining the inflammatory response in MASLD, although these pathways remain only partially understood. Furthermore, therapeutic options for MASLD and MASH are currently limited, with no approved pharmacological treatments available for the advanced stages of the disease. This review aims to provide a comprehensive overview of the current understanding of the cellular and molecular mechanisms involved in the inflammatory processes underpinning MASLD and MASH while also outlining the key challenges that lie ahead in the development of effective therapies.

## Introduction

### MASLD: A Global Health Challenge with Complex Metabolic and Inflammatory Roots

**Fig. 1:**
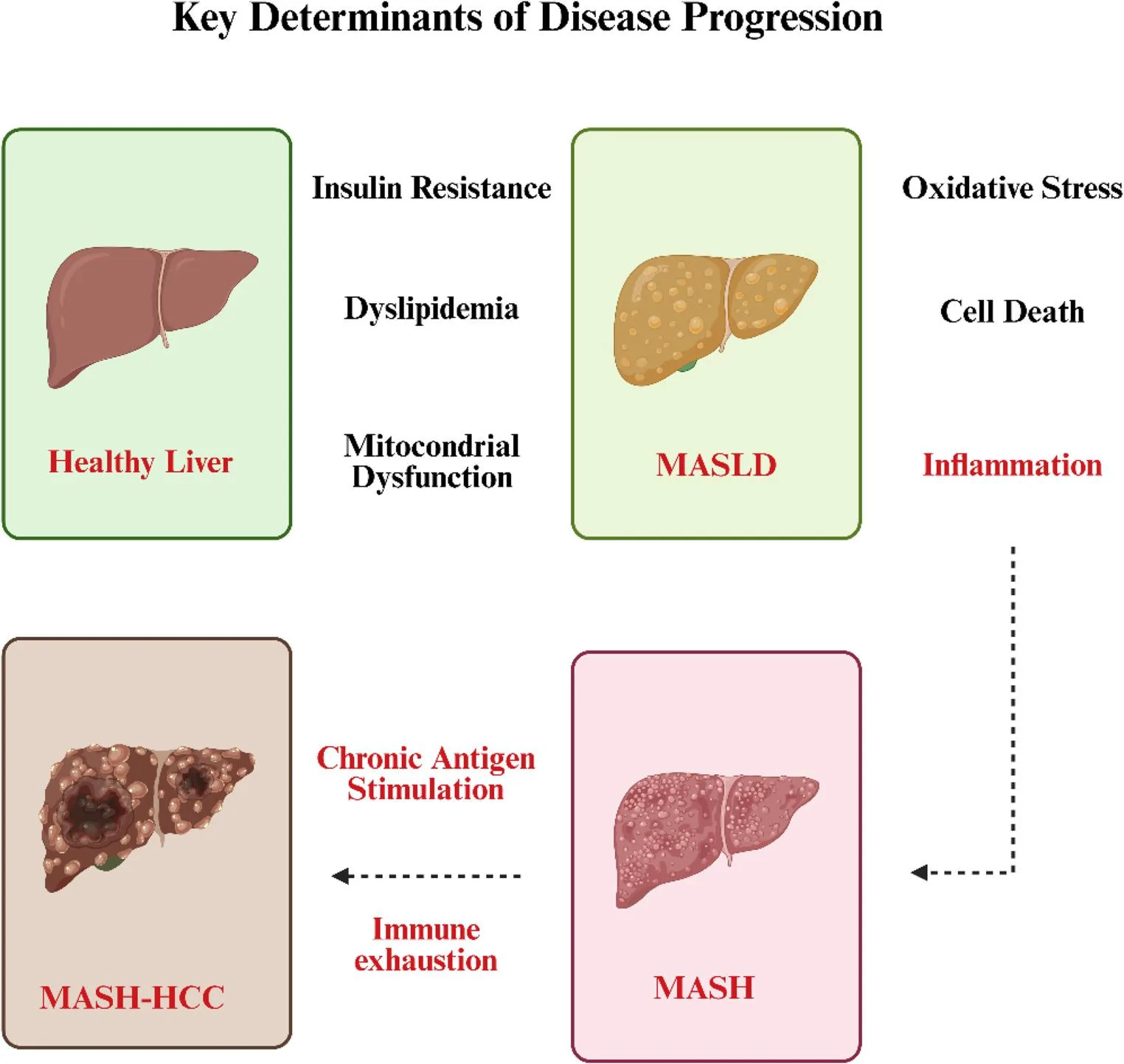
Natural history of MASLD and key determinants of disease progression. This conceptual overview illustrates the progression from a healthy liver to MASLD, MASH, and advanced stages, including hepatocellular carcinoma (HCC). Metabolic alterations such as insulin resistance, dyslipidemia, and mitochondrial dysfunction contribute to disease initiation, while oxidative stress, cell death, and inflammation promote progression. Persistent inflammatory and antigenic stimulation may further drive immune dysfunction and contribute to HCC development. Image created with BioRender.com

Metabolic dysfunction-associated steatotic liver disease (MASLD) has emerged as the most prevalent cause of chronic liver disease (CLD) globally, with a steadily increasing incidence that mirrors the worldwide rise in obesity [1]. The current obesity epidemic is multifactorial and driven by sedentary lifestyles, excessive intake of ultra-processed foods, and various socioeconomic determinants [2, 3]. Individuals with obesity frequently present with metabolic comorbidities such as insulin resistance (IR), type 2 diabetes (T2D), and dyslipidemia, all of which play central roles in MASLD pathogenesis [1]. Nonetheless, MASLD is not confined to obese individuals and may also occur in lean or metabolically healthy obese (MHO) individuals [4, 5]. MASLD encompasses a broad clinical spectrum, ranging from simple steatosis to more advanced forms, such as metabolic dysfunction-associated steatohepatitis (MASH). MASH is characterized by hepatic inflammation, hepatocellular injury, and varying degrees of fibrosis and is often accompanied by tissue remodelling and deposition of extracellular matrix (ECM) components. In its advanced stages, MASH may progress to cirrhosis and hepatocellular carcinoma (HCC), a progression collectively referred to as MASH-HCC [2]. The pathophysiological progression of MASLD involves multiple interconnected processes that are frequently initiated by IR. In individuals with obesity, chronic low-grade inflammation associated with adipose tissue expansion impairs insulin signalling, enhances lipolysis, and promotes the release of free fatty acids (FFAs) into the blood circulation [6]. These FFAs are subsequently delivered to the liver via the bloodstream, where they are esterified into triglycerides (TGs) [7]. Then, hepatocytes package TGs into very low-density lipoproteins (VLDLs), which are secreted and transported to peripheral tissues. However, when the influx of FFAs exceeds the liver’s metabolic capacity, surplus TGs accumulate within hepatocytes, leading to hepatic steatosis and cellular dysfunction [8]. In parallel, sustained FFA overload induces mitochondrial dysfunction through uncoupling of oxidative phosphorylation, thereby exacerbating oxidative stress [9]. These alterations collectively contribute to hepatocellular injury, apoptosis, and the activation of inflammatory pathways aimed at restoring tissue homeostasis [10]. These events are often accompanied by gut dysbiosis, which elevates circulating levels of lipopolysaccharide (LPS, also known as endotoxin), a condition known as endotoxemia that further amplifies hepatic inflammation [11]. Although the liver possesses remarkable regenerative potential, as famously illustrated by the myth of Prometheus (and Tityus), this capacity becomes progressively compromised under conditions of chronic injury, such as MASLD and MASH [12]. Ultimately, the replacement of damaged hepatocytes by ECM components, primarily collagen secreted by activated hepatic stellate cells (HSCs), results in fibrosis and, in advanced cases, cirrhosis that may progress to MASH-HCC (Fig. 1) [13]. Chronic inflammation represents a central driver of disease progression from simple steatosis to MASH and ultimately hepatocellular carcinoma (HCC). Persistent inflammatory signaling promotes hepatocellular injury, oxidative stress, and DNA damage, thereby fostering a mutagenic environment. In parallel, sustained antigen exposure leads to chronic immune activation, which over time may result in immune dysfunction and exhaustion. These processes collectively drive tissue remodeling and fibrogenesis while shaping a pro-tumorigenic microenvironment that supports HCC development. Thus, inflammation should not be viewed merely as a consequence of metabolic dysfunction but rather as a key mechanistic link that integrates metabolic stress with liver injury, fibrosis, and carcinogenesis [14]. While cirrhosis is a well-established risk factor for HCC, a substantial proportion of MASH-related HCC cases occur in the absence of cirrhotic changes, underscoring the intrinsic oncogenic potential of MASH [15]. Notably, with significant advances in the treatment of viral hepatitis, MASLD is now emerging as the leading global risk factor for HCC [16]. Epidemiological data indicate that MASLD affects nearly 38% of the global adult population, making it a major public health concern [17]. Its high prevalence, together with the frequent delay in diagnosis, underscores the urgent need for novel strategies to control inflammation, restore metabolic balance, and prevent progression to cirrhosis and HCC. At present, therapeutic options for MASLD and MASH remain limited, and no approved pharmacological treatments are available for advanced stages of the disease [18].

In this context, the present review aims to provide an overview of the current knowledge on the cellular and molecular mechanisms underlying the inflammatory processes driving the transition from MASLD to MASH. In particular, it seeks to highlight the complexity of the inflammatory pathways involved in MASLD progression, dissecting the key cellular and molecular components that orchestrate disease evolution. A comprehensive understanding of these mechanisms is crucial for the development of effective therapies capable of halting disease progression toward advanced stages.

### Mechanisms Driving Liver Inflammation in MASLD

The mechanisms that drive hepatic inflammation in MASLD involve a complex interplay of various cellular and molecular processes that change as the disease progresses. For clarity, the roles of different immune and non-immune cell populations, along with soluble mediators like cytokines, chemokines, and hepatokines, are discussed separately. However, it is important to note that this separation is only conceptual; in reality, these components function within the hepatic microenvironment as part of a highly interconnected and dynamically evolving network. These coordinated interactions shape the inflammatory landscape and contribute to the progression from MASLD to MASH.

Like many other organs, the liver harbors a population of tissue-resident macrophages known as Kupffer cells (KCs). These cells originate from erythro-myeloid progenitors in the yolk sac during embryogenesis and are maintained throughout adulthood via self-renewal mechanisms [19]. KCs reside within the liver sinusoids and are in close contact with endothelial cells, HSCs, and lymphocytes, where they continuously monitor the local environment and phagocytose potential pathogens or harmful debris [20].

In the context of MASLD, the accumulation of triglycerides (TGs) within hepatocytes leads to structural and functional alterations, ultimately resulting in cell injury and death [2]. Damaged hepatocytes release nuclear and cytoplasmic components collectively referred to as damage-associated molecular patterns (DAMPs), while gut-derived pathogen- and microbe-associated molecular patterns (PAMPs and MAMPs) translocate to the liver due to increased intestinal permeability (“leaky gut”), a condition frequently associated with MASLD.

In this setting, endocrine-disrupting chemicals (EDCs), including phthalates, bisphenols, per- and polyfluoroalkyl substances (PFAS), and organochlorine pesticides, together with heavy metals such as cadmium, arsenic, mercury, and lead, as well as emerging contaminants such as micro- and nanoplastics (MNPs), represent additional environmental drivers that may worsen disease progression. Heavy metals are increasingly recognized as contributors to MASLD progression, as they promote mitochondrial dysfunction, oxidative stress, lipid peroxidation, inflammasome activation, and epigenetic reprogramming, ultimately amplifying hepatic inflammation, immune dysregulation, and fibrogenesis. These environmental pollutants contribute to hepatic steatosis, inflammation, and fibrogenesis through multiple interconnected mechanisms. They interfere with nuclear receptor signaling pathways (e.g., PPARγ), promote gut microbiota dysbiosis and increased intestinal permeability, and impair mitochondrial and lysosomal function, ultimately disrupting lipid homeostasis. Notably, pollutant-induced barrier dysfunction enhances the translocation of PAMPs and MAMPs to the liver, amplifying inflammatory signaling. In parallel, these compounds can directly modulate hepatic immune responses by promoting Kupffer cell activation and cytokine production. Furthermore, pollutant-induced oxidative stress may increase DAMP release, thereby sustaining chronic inflammation [21–24].

Collectively, these signals activate innate immune responses and exacerbate hepatic inflammation via Toll-like receptor (TLR) engagement on Kupffer cells (KCs), triggering a pro-inflammatory transcriptional program [25].

Activated KCs produce a broad array of cytokines and chemokines that promote the recruitment and activation of circulating immune cells, particularly neutrophils and monocytes, thereby amplifying inflammation and contributing to tissue repair [26]. Recruited monocytes differentiate into monocyte-derived macrophages (MoMFs), which exhibit distinct phenotypes depending on the stage of the inflammatory response [27]. In the early stages, MoMFs acquire a pro-inflammatory phenotype, releasing cytokines, chemokines, reactive oxygen species (ROS), and proteolytic enzymes. Although these responses are intended to clear damage, they can actually aggravate tissue injury [28–30]. As the disease progresses, they shift toward a pro-resolving, tissue-reparative state characterized by the secretion of anti-inflammatory mediators such as interleukin-10 (IL-10) and annexin A1 (ANXA1) [28, 30–32]. They also produce pro-fibrogenic and remodelling molecules, including transforming growth factor-β1 (TGF-β1), osteopontin (OPN), galectin-3 (Gal-3), and matrix metalloproteinases (MMPs) [33–35]. Accordingly, MoMFs contribute to the activation of HSCs, driving their differentiation into α-SMA^+^ myofibroblast-like cells, which are responsible for collagen deposition and fibrotic progression [36, 37]. Additionally, ongoing liver injury and inflammation result in depletion of the resident KC pool. This niche is subsequently repopulated by monocyte-derived Kupffer cells (Mo-KCs), which adopt a reparative phenotype and support the resolution of inflammation and tissue remodelling [38]. While KCs have traditionally been viewed as central initiators of hepatic inflammation, emerging evidence suggests that several non-immune liver cell types also contribute significantly to this process. Hepatocytes, cholangiocytes, and HSCs, among others, have been shown to produce pro-inflammatory mediators that shape the immune landscape in MASLD and promote progression to MASH [39].

### Contribution of Liver-resident Non-immune Cells to MASLD-associated Inflammation

#### Hepatocytes

Steatotic hepatocytes are increasingly recognized as central players in initiating and perpetuating the inflammatory responses associated with MASLD. Lipid-laden hepatocytes undergo profound metabolic alterations that lead to inflammasome activation and the release of DAMPs. These DAMPs engage TLRs expressed on KCs, thereby triggering a cascade of inflammatory signalling events [40]. Inflammasome activation also sensitizes hepatocytes to lipopolysaccharide (LPS)-induced secretion of interleukin-1β (IL-1β), further stimulating myeloid cell activation and amplifying the pro-inflammatory cytokine milieu within the liver [40]. In addition, exposure of human hepatocytes to fatty acids (FAs) induces the expression of inflammatory cytokines and chemokines [41, 42], thereby sustaining immune cell recruitment from the circulation and reinforcing hepatic inflammation. In response to inflammatory cues, hepatocytes may aberrantly express class II major histocompatibility complex (MHCII) molecules, enabling antigen presentation to CD4⁺ T lymphocytes and contributing to the perpetuation of adaptive immune responses [43, 44]. In parallel, hepatocytes can produce interleukin-15 (IL-15), a cytokine that primes CD8⁺ cytotoxic T lymphocytes (CTLs) to acquire auto-aggressive features in the presence of metabolic stressors such as ATP and acetate. This enables CTLs to kill hepatocytes in an antigen-independent manner, thereby exacerbating steatohepatitis [45, 46]. IL-15 also promotes the hepatocyte-derived production of chemokines such as CCL2 and CCL5 and enhances the maturation and survival of natural killer (NK) and natural killer T (NKT) cells [47, 48]. Additionally, hepatocytes contribute to disease progression by secreting profibrogenic cytokines such as OPN and oncostatin M (OSM), which activate HSCs, promote ECM deposition, and drive fibrogenesis in chronic liver disease [49, 50]. Moreover, hepatocytes release a heterogeneous group of secretory proteins collectively termed hepatokines, including serpin B3 (SB3) and histidine-rich glycoprotein (HRG), which exert pro-inflammatory and pro-fibrogenic effects through multiple signalling pathways [51–53]. Some hepatocyte-derived factors may also contribute to hepatic carcinogenesis by fostering an immunosuppressive microenvironment and promoting tumor cell proliferation, survival, and invasiveness [50, 54, 55]. Taken together, these findings underscore thathepatocytes are not merely passive targets of lipotoxicity but also active contributors to the inflammatory and fibrotic processes that drive MASLD progression toward advanced disease stages.

#### Cholangiocytes

Cholangiocytes are a small population of epithelial cells that line the biliary tree and play crucial roles in bile synthesis and secretion [56]. However, their functional phenotype can shift significantly in response to tissue injury. Upon sensing damage signals through TLRs, cholangiocytes undergo transcriptional reprogramming that promotes cell proliferation as part of a reparative response [57]. In addition, cholangiocytes acquire pro-inflammatory properties, including the secretion of cytokines and chemokines, as well as the expression of adhesion molecules that mediate the recruitment and activation of immune cells [58]. Notably, cholangiocytes themselves become early targets of this pro-inflammatory program, which primarily affects the biliary epithelium and can subsequently contribute to hepatocellular injury [59]. During MASH, cholangiocytes proliferate and actively participate in liver inflammation and tissue remodelling through a well-characterized process known as the ductular reaction, which also involves hepatic progenitor cells (HPCs) and hepatocytes [60]. Furthermore, cholangiocytes can contribute to chronic inflammation by expressing MHC class II molecules and potentially activating T lymphocytes [61]. Thus, cholangiocytes play dual roles: on the one hand, they are essential for initiating tissue repair; on the other hand, they sustain hepatic inflammation, ultimately contributing to progressive tissue injury and liver failure [56].

#### Hepatic Stellate Cells

HSCs are liver-specific mesenchymal cells that reside in the perisinusoidal space (also known as the space of Disse), which is situated between the sinusoids and hepatocytes. Under physiological conditions, HSCs serve primarily as storage sites for vitamin A. However, following liver injury, HSCs lose their vitamin A-containing lipid droplets and transdifferentiate into myofibroblast-like cells, characterized by the expression of α-smooth muscle actin (α-SMA) and the secretion of elevated levels of pro-fibrogenic mediators and ECM components, thereby contributing to tissue scarring [62]. In addition to their central role in liver fibrogenesis, robust evidence indicates that HSCs also actively promote inflammatory responses [63, 64]. Once activated, HSCs both produce and respond to a wide range of pro-inflammatory mediators, including complement factors and cytokines, leading to the release of chemokines such as CCL2 and CXCL8 and other chemoattractants such as platelet-activating factor (PAF), which drive leukocyte recruitment and activation [63, 65]. Notably, HSCs are capable of sensing lipopolysaccharide (LPS) and activating the inflammasome, providing an additional mechanism by which they can contribute to hepatic inflammation [66]. Upon activation, HSCs also upregulate adhesion molecules such as intercellular adhesion molecule-1 (ICAM-1), vascular cell adhesion molecule-1 (VCAM-1), and neural cell adhesion molecule (NCAM/CD56), which bind leukocyte integrins and facilitate their transmigration and infiltration into the liver parenchyma [63]. Furthermore, HSCs express hematopoietic growth factors such as macrophage colony-stimulating factor (M-CSF) and stem cell factor (SCF), promoting the proliferation and differentiation of hematopoietic precursors and amplifying the inflammatory cascade [63, 67]. HSCs can also act as professional antigen-presenting cells (APCs) capable of processing lipid and protein antigens and expressing MHC class II molecules during inflammation [65]. Taken together, these findings suggest that HSCs contribute to hepatic inflammation through multiple mechanisms, exacerbating steatohepatitis. However, the picture is more complex: HSCs also produce anti-inflammatory (e.g., interleukin-10 (IL-10)) and immunomodulatory (e.g., programmed death-ligand 1 (PDL-1)) molecules [63, 65]. Moreover, HSCs can promote the expansion of both monocytic and polymorphonuclear myeloid-derived suppressor cells (M-MDSCs and PMN-MDSCs) via CD44-mediated signalling pathways [68]. These data support the view that, similar to immune cells such as macrophages, HSCs display functional plasticity that is modulated by cues from the surrounding tissue microenvironment [67, 69].

#### Liver Sinusoidal Endothelial Cells (LSECs)

Liver sinusoidal endothelial cells (LSECs) are central players in initiating hepatic inflammatory responses. These specialized endothelial cells line the liver sinusoids, forming a highly permeable barrier that facilitates the exchange of gases, nutrients, and metabolites. Under physiological conditions, LSECs maintain close contact with circulating blood cells, HSCs, and hepatocytes [70–72]. Beyond their barrier function, LSECs perform a variety of additional roles. Notably, they regulate blood pressure by releasing vasodilatory mediators such as nitric oxide (NO) in response to shear stress [71–73]. Moreover, LSECs help maintain HSCs in a quiescent state by inhibiting their trans-differentiation into contractile α-SMA⁺ myofibroblast-like cells [70]. When HSCs become activated, they contribute to increased sinusoidal vascular resistance, thereby increasing intrahepatic blood pressure, a key feature of liver cirrhosis that can lead to further complications such as ascites and variceal bleeding [74, 75]. LSECs also exhibit remarkable endocytic capacity, which is mediated by the expression of multiple surface receptors, including Fc gamma receptor IIb (FcγRIIb), which enables the clearance of immune complexes [76, 77]. However, exposure to toxic lipids can impair LSEC function, leading to endothelial dysfunction, disruption of the hepatic microvascular barrier, heightened inflammatory signalling, and, ultimately, the progression of liver injury and fibrosis [78, 79]. In the context of MASH, LSECs sustain hepatic inflammation by producing chemokines and cytokines and aberrantly expressing adhesion molecules such as VCAM-1, ICAM-1, and VAP-1. These changes promote leukocyte activation and recruitment, thereby perpetuating liver inflammation and disease progression [80].

### Soluble Mediators in MASLD-driven Liver Inflammation

#### Cytokines

Cytokines play pivotal roles in the pathogenesis of metabolic steatohepatitis (MASH), acting as central mediators of hepatic inflammation, immune cell activation, and fibrogenesis [81]. In response to metabolic stress and the accumulation of toxic lipids, both resident and infiltrating immune cells, including hepatocytes, KCs, HSCs, and LSECs, release a broad spectrum of pro-inflammatory cytokines, such as TNF-α, IL-6, IL-1β, and TGF-β [82, 83]. These mediators promote leukocyte recruitment and activation, amplify local inflammation, and drive fibrotic remodelling through the activation of HSCs [84]. Specifically, TNF-α and IL-1β contribute to insulin resistance and oxidative stress, exacerbating hepatocellular injury, whereas TGF-β functions as a potent inducer of fibrogenic transformation [85]. In addition to these classical cytokines, other immune mediators, such as interleukin‑15 (IL‑15), interleukin‑17 (IL‑17), and OPN, have emerged as key contributors to MASH pathogenesis [48, 86, 87]. IL‑15 promotes liver inflammation by activating NK cells and memory CD8⁺ T cells, promoting cytotoxicity and immune infiltration [48]. IL‑17, which is secreted predominantly by Th17 cells, acts synergistically to induce the release of inflammatory mediators from hepatocytes, KCs, and HSCs, thereby amplifying inflammation and stimulating fibrogenesis through neutrophil recruitment and HSC activation [86]. OPN, a multifunctional cytokine-like glycoprotein, is overexpressed in hepatocytes and immune cells in MASH and contributes to chronic hepatic inflammation and fibrosis [88]. It enhances macrophage recruitment and polarization toward a pro-inflammatory phenotype while directly promoting HSC activation and extracellular matrix deposition [89, 90]. Overall, persistent immune activation creates a pro-inflammatory and -fibrotic microenvironment that drives the transition from simple steatosis to advanced fibrosis, cirrhosis, and HCC [91]. Targeting these cytokines and their downstream pathways represents a promising therapeutic avenue to mitigate hepatic inflammation and slow disease progression to MASH.

#### Chemokines

Chemokines are key regulators of immune cell trafficking and play a fundamental role in the pathogenesis and progression of MASH [92]. In response to metabolic and inflammatory factors, various liver-resident and non-resident cells upregulate the expression of chemokines such as CCL2, CCL5, and CXCL10 [93–95]. These chemokines bind to their respective receptors (e.g., CCR2 for CCL2 and CCR5 for CCL5) expressed on circulating immune cells, mediating their recruitment into the hepatic parenchyma [96, 97]. This chemotactic migration represents a critical step in amplifying liver inflammation, as recruited monocytes differentiate into pro-inflammatory macrophages and monocyte-derived dendritic cells (moDCs), which perpetuate inflammatory signalling and tissue injury [28, 98, 99]. Among these chemokines, CCL2 has been extensively characterized because of its role in monocyte/macrophage recruitment, and elevated CCL2 levels are correlated with disease severity and contribute to HSC activation [100]. In parallel, CCL5 and CXCL10 recruit lymphocyte subsets, including T cells and NK cells, further aggravating hepatocellular damage [92, 101]. Furthermore, fractalkine (CX3CL1), a membrane-bound and soluble chemokine primarily produced by hepatic parenchymal and non-parenchymal cells, plays a distinct role by binding to its unique receptor CX3CR1 expressed on monocytes, NK cells, and monocyte-derived dendritic cells [102–104]. The CX3CL1/CX3CR1 axis is crucial for the recruitment, retention, and survival of CX3CR1⁺ monocytes and moDCs within the inflamed liver [99, 102, 103]. In MASH, moDCs contribute to the amplification of the inflammatory response by producing pro-inflammatory cytokines and presenting antigens to T cells, promoting adaptive immune activation and fibrogenesis [99, 105, 106]. Genetic or pharmacologic disruption of the CX3CL1/CX3CR1 pathway has been shown to reduce hepatic inflammation in preclinical models, underscoring its pathogenic relevance and therapeutic potential [99, 103]. However, the precise contribution of the CX3CL1/CX3CR1 axis to liver damage remains a matter of debate [103, 107–109]. Overall, the chronic production of chemokines, including CCL2, CCL5, CXCL10, and CX3CL1, and the persistent recruitment of immune cells create a self-perpetuating vicious cycle of inflammation, hepatocellular injury, and fibrotic remodelling that defines MASH progression [110].

#### Hepatokines

**Fig. 2:**
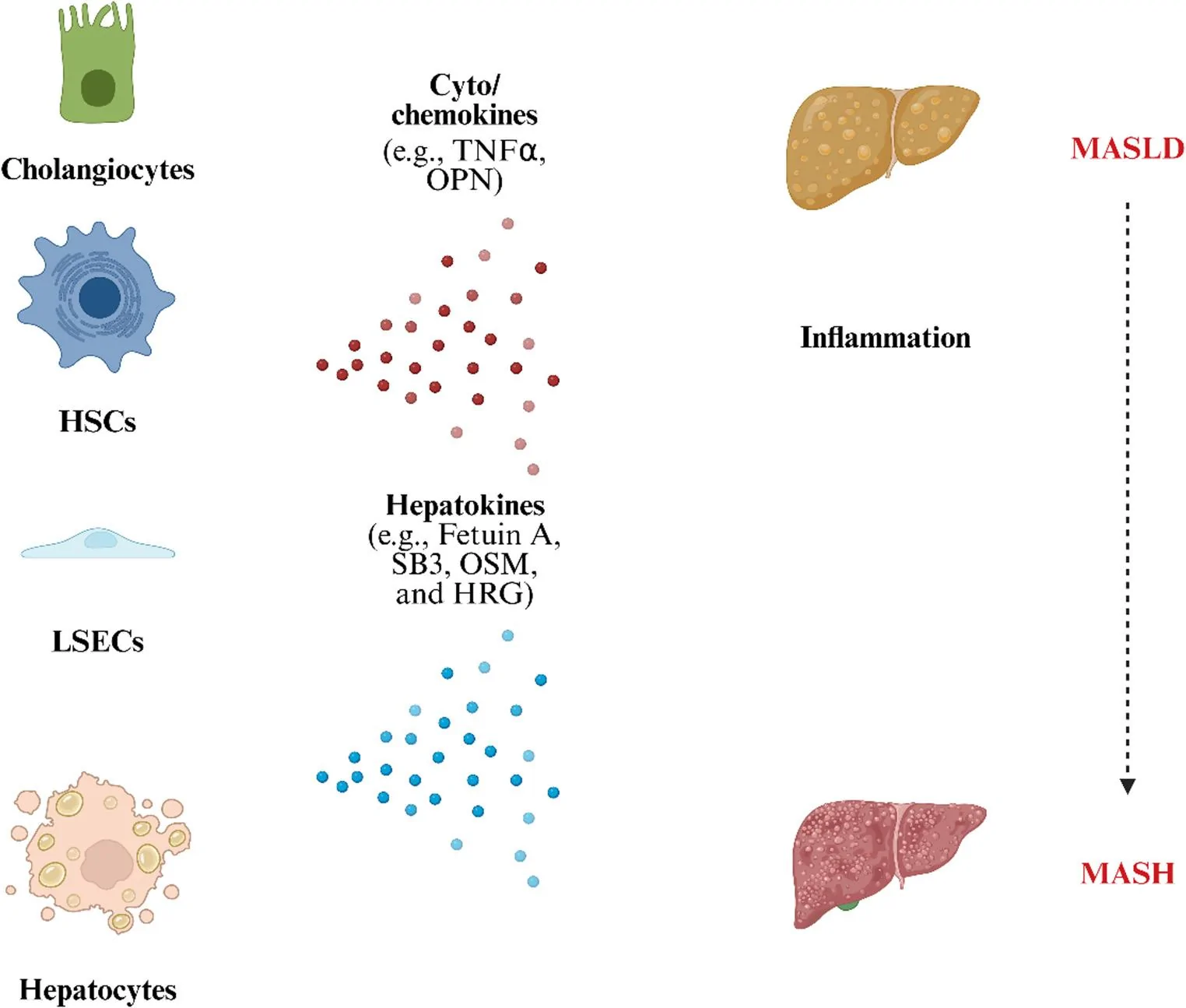
Liver-resident cells and molecular mediators in MASLD-to-MASH progression

Hepatokines are liver-derived secretory proteins that play a central role in the regulation of systemic metabolism, inflammation, and inter-organ communication [111]. In the context of MASH, several hepatokines, including fetuin-A, fibroblast growth factor 21 (FGF21), and angiopoietin-like protein 8 (ANGPTL8), have been implicated in disease progression [112]. Fetuin-A, which is upregulated in insulin-resistant states, contributes to hepatic inflammation by promoting TLR4-mediated activation of immune cells, thereby exacerbating liver injury [113, 114]. In contrast, FGF21 is generally considered hepatoprotective, as it promotes fatty acid oxidation and suppresses inflammatory pathways; however, its circulating levels are often elevated in MASH, likely reflecting a compensatory response to metabolic stress [115, 116]. Similarly, ANGPTL8, a regulator of lipid metabolism, is overexpressed in steatohepatitis and may contribute to hepatic lipid accumulation and inflammation. However, owing to conflicting findings, its role in MASLD warrants further investigation [117]. In addition to these classical hepatokines, other liver-derived mediators, such as Serpin B3 (SB3), histidine-rich glycoprotein (HRG), and oncostatin M (OSM), are gaining attention for their roles in modulating inflammation and fibrosis in MASH [37, 51–53]. SB3, a serine protease inhibitor induced in damaged hepatocytes, has been shown to counteract oxidative stress, inhibit hepatocyte apoptosis, and promote epithelial‒mesenchymal transition (EMT), ultimately contributing to fibrosis and the development of hepatocellular carcinoma (HCC) [51, 55, 118, 119]. HRG, a multifunctional glycoprotein involved in immune regulation and tissue remodelling, has altered expression in MASH and may influence macrophage polarization and ECM turnover, thereby perpetuating chronic liver inflammation and contributing to cancer development [53, 54, 120]. OSM, a cytokine of the IL-6 family, is markedly upregulated in fibrotic livers and promotes HSC activation and collagen production, reinforcing the inflammatory and fibrotic milieu characteristic of MASH [37, 50, 121–123] (Fig. 2).

Together, these liver-derived mediators constitute a complex signalling network that links metabolic dysregulation with inflammation and fibrosis. The emerging roles of these genes in MASH pathophysiology not only increase our understanding of disease mechanisms but also provide promising targets for biomarker discovery and therapeutic intervention.

Altogether, these findings highlight the progressive reorganization of immune responses during disease evolution, where cellular and molecular components operate as an integrated network rather than as isolated entities, ultimately sustaining hepatic inflammation and advancing disease progression.

This conceptual overview illustrates the contribution of liver-resident cells to inflammation during the transition from MASLD to MASH. Cholangiocytes, hepatocytes, hepatic stellate cells (HSCs), and liver sinusoidal endothelial cells (LSECs) shape a pro-inflammatory microenvironment through the release of cytokines, chemokines, and hepatokines. These mediators promote immune cell recruitment and activation, sustaining hepatic inflammation and disease progression. Image created with BioRender.com.

### Innate Immunity in MASLD-associated Inflammation

#### Neutrophils

Neutrophils are increasingly recognized as active contributors to the pathogenesis of MASH rather than passive bystanders. Early neutrophil infiltration, as evidenced by elevated myeloperoxidase (MPO) activity and neutrophil elastase release, has been observed in murine models of MASH and is correlated with the severity of hepatocellular injury. Pharmacological inhibition of neutrophil activity via the use of anti-Ly6G antibodies or neutrophil elastase inhibitors such as sivelestat significantly attenuates hepatic inflammation and injury in dietary MASH models [124–127]. A key mechanism by which neutrophils exacerbate liver pathology is the formation of neutrophil extracellular traps (NETs). NETs have been detected in liver biopsies from MASH patients, with 94.1% of cases showing NETs decorated with IL-1β and IL-17 A, which correlate strongly with histological disease severity [128]. In murine MASH models, both genetic ablation of NETosis (in PAD4⁻/⁻ mice) and pharmacological degradation of NETs via DNase I lead to a reduction in immune cell infiltration, fibrogenesis, and progression to HCC [129]. Mechanistically, NETs promote HSC activation via the TLR3 and COX-2 signalling pathways, inducing metabolic reprogramming and enhancing fibrogenic activity [130]. Additionally, NETs contribute to the establishment of a pro-thrombotic hepatic microenvironment by promoting thrombin and fibrin deposition, which damages sinusoidal endothelial cells, a process that can be mitigated by DNase I administration [131]. Together, these findings underscore the role of neutrophils, particularly through NET formation, as central effectors of hepatic inflammation, fibrogenesis, and MASH-associated carcinogenesis and position NETs as attractive therapeutic targets.

#### Monocytes and Macrophages

Monocytes and macrophages play pivotal roles in driving the inflammatory milieu and fibrogenic processes that characterize metabolic steatohepatitis (MASH) [132]. In response to hepatocellular injury and lipid accumulation, damaged hepatocytes and activated Kupffer cells release chemokines, particularly CCL2, that recruit circulating CCR2⁺ monocytes to the liver [133]. Upon recruitment, these monocytes differentiate into pro-inflammatory macrophages that secrete high levels of cytokines, such as TNF-α, IL-1β, and IL-6, thereby amplifying local inflammation and promoting hepatocyte apoptosis [32, 134]. At the molecular level, macrophage activation is driven by pathogen-associated molecular patterns (PAMPs) and DAMPs, which are recognized by pattern recognition receptors (PRRs), such as TLRs and NOD-like receptors (NLRs) [134]. For example, the engagement of TLR4 by lipopolysaccharide (LPS) and saturated fatty acids activates the NF-κB signalling pathway, leading to the transcription of pro-inflammatory genes [32, 135, 136]. Moreover, activation of the NLRP3 inflammasome in macrophages induces caspase-1-mediated maturation and the release of IL-1β and IL-18, further exacerbating hepatic inflammation [83, 137]. The macrophage population in MASH is highly heterogeneous and plastic [138]. Classically activated macrophages promote inflammation and tissue injury. Conversely, alternatively activated macrophages are involved in tissue remodelling and fibrosis through the secretion of anti-inflammatory cytokines such as IL-10 and TGF-β [35, 139, 140]. In MASH, persistent metabolic and inflammatory stimuli promote a sustained pro-fibrotic macrophage phenotype that activates HSCs via TGF-β and platelet-derived growth factor (PDGF), leading to extracellular matrix deposition and fibrosis [141, 142]. KCs also undergo phenotypic shifts in MASH. Lipid overload and gut-derived endotoxins stimulate KCs to produce reactive oxygen species (ROS) and inflammatory cytokines, exacerbating hepatocyte injury and enhancing monocyte recruitment [143, 144]. Therefore, targeting key molecular pathways involved in monocyte recruitment (e.g., the CCR2/CCL2 axis), macrophage polarization, and inflammasome activation represents a promising therapeutic strategy to mitigate inflammation and fibrosis in MASH, positioning macrophages as a central focus of ongoing research.

#### Dendritic Cells

Dendritic cells (DCs) are professional antigen-presenting cells that play a complex role in bridging innate and adaptive immune responses during MASH [145]. In the liver, DCs capture and process antigens and DAMPs released from injured hepatocytes, leading to their activation and maturation [146]. Activated DCs produce pro-inflammatory cytokines such as IL-12 and TNF-α, promoting the differentiation and activation of T helper (Th) cells and thereby exacerbating liver inflammation [147, 148]. Additionally, DCs contribute to the recruitment and activation of other immune cells, including neutrophils and macrophages, amplifying the hepatic inflammatory response [149]. Studies have demonstrated that hepatic DCs in MASH exhibit a pro-inflammatory phenotype with increased expression of costimulatory molecules and a reduced tolerogenic capacity, which sustains chronic inflammation and fibrosis progression [99, 103, 145]. Conversely, tolerogenic DC subsets may play protective roles by promoting regulatory T-cell responses and limiting immune-mediated liver damage [150]. The dynamic balance between inflammatory and regulatory DC functions is therefore critical in MASH pathogenesis, making these cells potential targets for therapeutic modulation.

#### Platelets

Platelets, which are traditionally recognized for their role in hemostasis and thrombosis, are now acknowledged as key immune and inflammatory effectors [151]. They interact dynamically with endothelial and immune cells, releasing a wide array of cytokines, chemokines, and growth factors that regulate vascular integrity and inflammation. Emerging evidence indicates that platelets act as sentinels linking coagulation and innate immunity, thereby contributing to inflammatory responses in several metabolic and cardiovascular diseases [152, 153]. In the context of MASLD, platelets play a pivotal role in initiating and perpetuating hepatic inflammation [154]. Platelet activation in MASLD is driven by multiple metabolic and inflammatory stimuli, including oxidative stress, insulin resistance (IR), elevated free fatty acids (FFAs), and gut-derived endotoxins that activate Toll-like receptor 4 (TLR4) on platelet membranes [155, 156]. These factors increase platelet sensitivity to agonists such as ADP, thrombin, and collagen, resulting in increased aggregation and secretion of pro-inflammatory mediators. In addition, the altered lipid composition of platelet membranes and exposure to circulating cytokines (e.g., TNF-α and IL-6) further potentiate platelet activation [157]. Once activated, platelets adhere to the liver sinusoidal endothelium and release pro-inflammatory mediators such as TGF-β, PDGF, and IL-1β, which promote leukocyte recruitment and hepatic stellate cell activation [153]. Platelet–leukocyte aggregates further amplify liver injury by stimulating neutrophil extracellular trap (NET) formation and activating Kupffer cells, thereby reinforcing the pro-inflammatory microenvironment [157]. Under conditions of metabolic dysfunction, platelet hyperreactivity and procoagulant activity contribute to endothelial activation, microthrombus formation, and local hypoxia, all of which exacerbate hepatic inflammation [158]. Moreover, recent findings suggest that platelets potentiate inflammasome activation in both human macrophages and neutrophils, leading to increased IL-1β production and underscoring their role as active participants in innate immune activation [155]. Overall, platelets emerge not only as markers of disease progression but also as active drivers of hepatic inflammation in MASLD. Therefore, targeting platelet activation or their immune signalling pathways may represent a promising therapeutic strategy to mitigate inflammation and fibrosis in metabolic liver disease.

#### Natural Killer T (NKT) Cells

NKT cells are a distinct subset of innate-like T lymphocytes that co-express T-cell receptors and natural killer cell markers and are uniquely abundant in the liver [159]. In MASLD, emerging evidence indicates that NKT cells participate in both the initiation and progression of disease by sensing lipid antigens presented by CD1d-expressing hepatic antigen-presenting cells; releasing cytokines such as IFN-γ, IL-4, and IL-17; and interacting with other immune and non-immune hepatic cells [160]. Studies in patients with advanced fatty liver disease have shown that increased intrahepatic NKT cell numbers are correlated with disease severity, steatohepatitis, and fibrosis [161]. Mechanistically, activation of the hedgehog (Hh) signalling pathway in injured liver tissue promotes the recruitment and retention of NKT cells via chemokines such as CXCL16, which, in turn, amplify fibrogenic responses by driving hepatic stellate cell activation and myofibroblast transformation [161]. Interestingly, the role of NKT cells appears to be highly context dependent. In the early stages of disease, these cells may exert regulatory functions, whereas in more advanced stages, they acquire a pro-inflammatory and pro-fibrotic phenotype, releasing cytokines and chemokines that promote the recruitment of neutrophils and macrophages and activate hepatic stellate cells [161–163]. A key molecular modulator of this process is the NF-κB1/p50 subunit, which acts as a transcriptional regulator that dampens excessive inflammatory activation in the liver. Experimental models have demonstrated that p50 deficiency enhances hepatic inflammation and fibrosis through uncontrolled NKT cell activation. In p50^-^deficient mice fed a methionine- and choline-deficient (MCD) diet, researchers reported increased hepatic infiltration of NKT cells, elevated IFN-γ and OPN levels, and accelerated progression toward steatohepatitis and fibrosis compared with those in wild-type controls [164]. These findings suggest that NF-κB1/p50 normally restrains NKT-cell-mediated inflammatory cascades, maintaining hepatic immune tolerance. In MASLD, dysregulated NF-κB1/p50 signalling, potentially triggered by metabolic stress, lipotoxicity, or oxidative injury, may lead to excessive activation and retention of NKT cells, amplifying pro-inflammatory and pro-fibrogenic pathways [164]. Overall, NKT cells have emerged not only as key modulators of liver immunity in MASLD but also as potential therapeutic targets to modulate the transition from simple steatosis to steatohepatitis and fibrosis.

### T Cells and Beyond: Adaptive Immunity as a Driver of MASH

#### T cells

T cells are key mediators of adaptive immunity and play a central role in the pathogenesis of MASH. Both CD4⁺ helper and CD8⁺ cytotoxic T cells infiltrate the liver during disease progression, contributing to inflammation, hepatocyte injury, and fibrosis [165, 166]. CD4⁺ T cells differentiate into the pro-inflammatory subsets Th1 and Th17 cells, which secrete cytokines, including interferon-gamma (IFN-γ), TNF-α, and interleukin-17 (IL-17) [167–169]. These cytokines amplify hepatic inflammation, promote hepatocyte apoptosis, and stimulate HSCs, driving fibrogenesis [170]. The Th17 subset is particularly implicated in sustaining chronic inflammation and recruiting neutrophils, further exacerbating tissue damage [171, 172]. Regulatory T cells (Tregs), which normally suppress excessive immune activation and maintain immune tolerance, are often functionally impaired or reduced in individuals with metabolically unhealthy obesity (MUO) and MASH, leading to unchecked inflammatory responses [173–175]. However, emerging evidence suggests that Tregs may also exacerbate hepatic injury, promote fibrosis, and contribute to hepatocarcinogenesis [176–179]. Therefore, Tregs appear to play multifaceted roles in MASH, which may shift throughout disease progression; however, their precise contribution remains a matter of ongoing debate.

In the context of MASH, CD8⁺ T cells that exhibit auto-aggressive properties are of particular interest, as they recognize stressed or altered hepatocytes as targets and induce their death independently of specific antigen stimulation. These autoreactive CD8⁺ T cells mediate direct cytotoxicity through the FAS/FAS ligand pathway, leading to hepatocyte apoptosis and increasing liver injury. In addition, they secrete pro-inflammatory cytokines such as TNF-α, thereby contributing to the inflammatory milieu and the progression of fibrosis [46]. The activation and expansion of these pathogenic CD8⁺ T cells may be driven by IL-15 produced by hepatocytes under metabolic stress conditions, such as lipotoxicity and oxidative damage [46]. This self-directed immune response exacerbates tissue injury and sustains the chronic inflammatory environment characteristic of MASH. Interestingly, the administration of selective sodium-glucose co-transporter 2 (SGLT2) inhibitors, such as empagliflozin (EMPA), has been shown to reduce the hepatic recruitment of auto-aggressive CD8⁺ T cells and ameliorate liver injury in both murine models and human patients [180]. In addition, recent findings have described the presence of clonal expansion of CD8⁺ T cells in MASH livers. These cells also express markers of exhaustion, which are typically associated with chronic antigenic stimulation through the T-cell receptor (TCR). Taken together, these results suggest that the accumulation of CD8⁺ T cells in the MASH liver may be driven not only by cytokine and metabolic stimuli but also by antigen-dependent activation and clonal expansion [166]. However, further investigations are needed to identify the specific antigens responsible for CD8⁺ T-cell clonal expansion. It is conceivable that oxidative stress-derived epitopes (OSEs), previously implicated in CD4⁺ T-cell activation [167], may also play a role in this context.

Furthermore, T cells interact closely with other immune populations, such as macrophages and dendritic cells, which secrete cytokines, such as IL-12 and IL-23 that promote the differentiation of pro-inflammatory T-cell subsets, thereby reinforcing a deleterious inflammatory feedback loop [181, 182]. Overall, the imbalance between pro-inflammatory T-cell responses and regulatory mechanisms is a key driver of MASH pathogenesis, highlighting the potential of T-cell-targeted immunotherapies to mitigate liver inflammation and fibrosis.

#### B lymphocytes

B lymphocytes have emerged as important players in the immunopathogenesis of MASH [183, 184]. They accumulate in the liver during disease progression and contribute to inflammation and fibrosis through various mechanisms [185–187]. Activated B cells produce pro-inflammatory cytokines such as IL-6 and TNF-α, which promote hepatic inflammation and activate other immune cells, including T cells and macrophages [183, 187–189]. Furthermore, B cells act as antigen-presenting cells, sustaining adaptive immune responses within the hepatic microenvironment [190, 191]. Importantly, B cells recognize oxidative stress-derived neoantigens, such as malondialdehyde (MDA)-modified proteins, leading to the production of pathogenic autoantibodies [167, 183, 192]. These antibodies form immune complexes that likely activate complement pathways, further exacerbating liver injury and fibrosis [184, 192–194]. In support of this, experimental depletion or functional impairment of B cells in murine models results in reduced hepatic inflammation and fibrosis, underscoring their pathogenic role in MASH [183, 194, 195]. Additionally, regulatory B cells (Bregs), which produce IL-10 and contribute to the maintenance of immune tolerance, are often reduced or functionally impaired in MASH, resulting in an imbalance that favours chronic inflammation [187, 194]. Although the multifaceted role of B cells in promoting inflammation and fibrogenesis remains to be fully elucidated, they represent promising targets for therapeutic intervention in MASH.

#### Bidirectional Crosstalk Between Innate and Adaptive Immunity in MASH

**Fig. 3:**
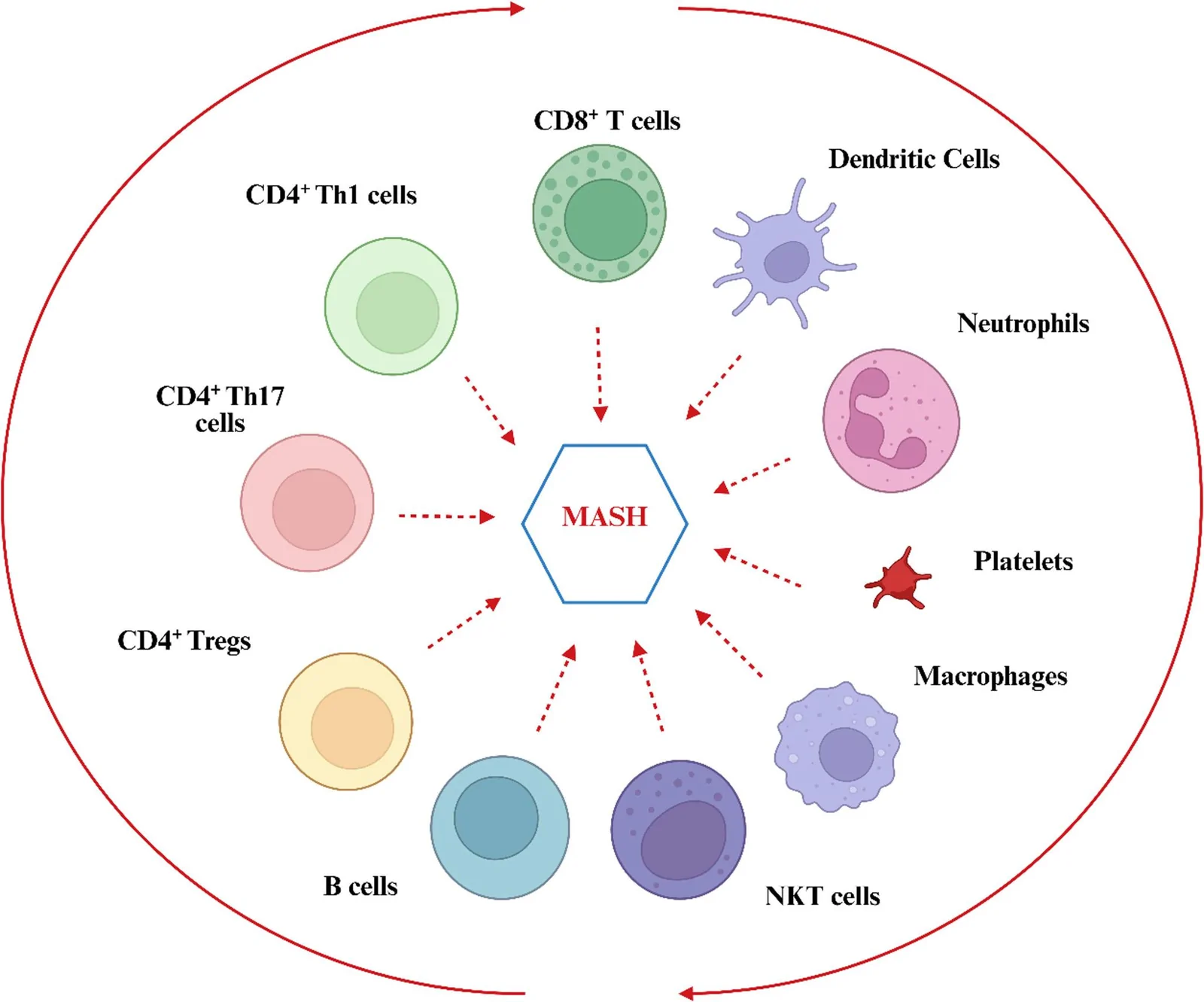
Innate and adaptive immune cell interactions in MASH. This conceptual overview illustrates the bidirectional crosstalk between innate and adaptive immune cells in MASH. Innate immune cells promote the recruitment and activation of adaptive immune responses, while adaptive immune cells reciprocally modulate innate cell functions, establishing a self-sustaining inflammatory network that contributes to disease progression. Image created with BioRender.com

The progression of MASH involves a complex interplay between innate and adaptive immune responses, where bidirectional crosstalk critically shapes disease outcomes [163, 196]. As mentioned above, innate immune cells such as KCs, LSECs, and DCs detect danger signals, including lipotoxicity, oxidative stress, and DAMPs, leading to the secretion of pro-inflammatory cytokines (e.g., TNF-α and IL-1β) and chemokines that orchestrate immune cell recruitment and activation [39, 163]. This inflammatory milieu promotes the activation and differentiation of adaptive immune cells, including T and B lymphocytes, which further amplify and sustain hepatic inflammation. In addition, adaptive immune cells modulate innate cell functions; for example, CD4⁺ T helper cells produce IFN-γ, which enhances macrophage activation, while Tregs can suppress excessive innate immune responses, promoting the resolution of inflammation [165, 175]. Additionally, antigen-presenting cells such as dendritic cells bridge innate and adaptive immunity by processing and presenting liver-derived antigens, leading to antigen-specific T-cell responses that perpetuate tissue damage or fibrosis [169]. The dynamic and reciprocal crosstalk among these immune compartments underpins the chronic inflammatory milieu characteristic of MASH and may constitute a promising target for therapeutic interventions aimed at re-establishing immune homeostasis (Fig. 3).

#### Antigenic Triggers in MASH: Unveiling Immune Activation

**Fig. 4:**
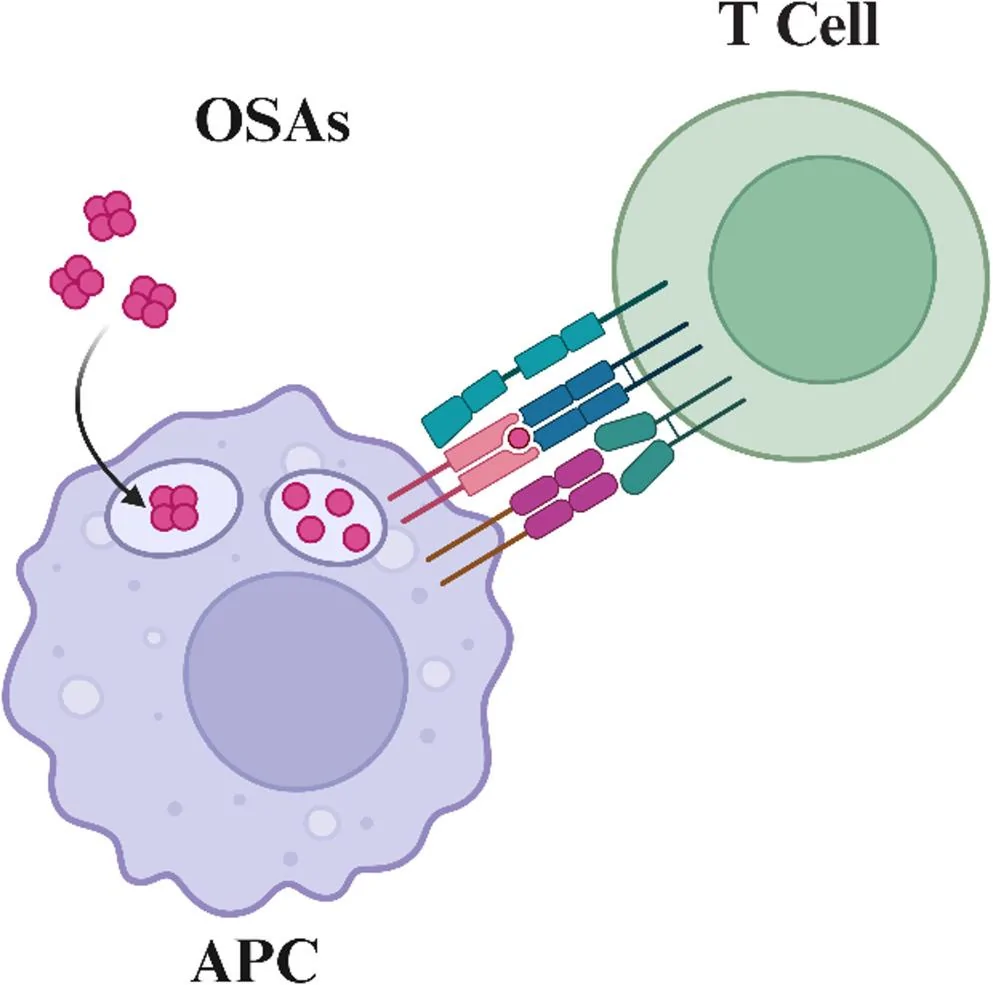
Antigen presentation and co-stimulatory signaling in APC–T cell crosstalk in MASH. This conceptual overview illustrates interactions between antigen-presenting cells (APCs) and T cells in MASH-associated inflammation. APCs present oxidative stress–derived antigens (OSAs) to T cells, contributing to their activation. Full T-cell activation requires additional co-stimulatory signals (e.g., ICOS/ICOSL, CD40/CD40L, OX40/OX40L), which represent general mechanisms of T-cell activation. Image created with BioRender.com

In MASH, immune activation is driven not only by PAMPs but also by self-derived antigens arising from liver injury and oxidative stress [185, 191, 194]. Among these, oxidative stress-induced neoantigens play a pivotal role in promoting hepatic inflammation (Fig. 4). Specifically, lipid peroxidation products such as malondialdehyde (MDA) and 4-hydroxynonenal (4-HNE) covalently modify cellular proteins, generating novel epitopes that are recognized as non-self by the immune system, thereby eliciting adaptive immune responses [167, 192, 197]. In addition, oxidative stress promotes post-translational modifications, such as carbonylation and nitration, that increase the immunogenicity of endogenous proteins [192, 198, 199]. These oxidative stress-derived epitopes activate both T and B lymphocytes, thereby sustaining chronic hepatic inflammation and contributing to tissue damage [167, 197]. Concurrently, damaged hepatocytes release DAMPs, such as high-mobility group box 1 (HMGB1) and ATP, which engage innate immune receptors, including Toll-like receptors (TLRs) and inflammasomes, on KCs and DCs. This activation amplifies inflammatory signalling and antigen presentation, establishing a self-perpetuating loop that exacerbates hepatic injury [200]. As a result, the sustained presentation of stress-induced antigens by antigen-presenting cells (APCs) drives lymphocyte activation, breaks immune tolerance, and promotes progression from MASH to fibrosis and cirrhosis. A deeper understanding of these antigen-driven immune mechanisms may reveal novel immunomodulatory targets to alleviate chronic inflammation and prevent liver damage.

#### Costimulatory Molecules in MASH

The dynamic and reciprocal crosstalk between innate and adaptive immunity underscores the critical role of costimulatory molecules, including their soluble isoforms, in the pathogenesis of MASH [98, 196, 201]. These molecules act as key regulators of immune activation and are expressed primarily by APCs, such as DCs and macrophages [98, 146, 202]. They deliver indispensable secondary signals required for the full activation, proliferation, and survival of T lymphocytes [98]. In addition, costimulatory pathways enhance T-cell effector functions, including cytokine production and clonal expansion, thereby sustaining and amplifying immune responses [203, 204]. In the context of MASH, the upregulation of these pathways within the hepatic microenvironment has been observed, facilitating sustained T-cell activation and chronic inflammatory responses [169, 205, 206] (Fig. 4). In parallel, inhibitory costimulatory molecules such as programmed death-ligand 1 (PD-L1) are also elevated, likely as a compensatory mechanism to restrain excessive immune activation [207–209]. Paradoxically, this may contribute to immune cell exhaustion and impaired tissue repair, thereby exacerbating disease progression [210, 211]. The resulting imbalance between activating and inhibitory costimulatory signals promotes T-cell responses and reinforces the proinflammatory loop between innate and adaptive immune cells, thereby driving fibrogenesis and advancing MASH pathology [169, 206]. Consequently, therapeutic modulation of costimulatory pathways holds promise as a strategy to reestablish immune homeostasis and mitigate liver injury in MASH.

#### ICOS/ICOS-L Axis

The inducible T-cell co-stimulator (ICOS) receptor is expressed primarily on activated T cells, whereas its ligand, ICOSL (also known as B7-H2 or CD275), is found on APCs, including DCs, macrophages, and B cells, and in several types of non-immune cells [212]. This costimulatory axis plays a pivotal role in regulating T-cell differentiation, survival, and cytokine secretion, thereby critically influencing the balance between effector T cells and regulatory T cells (Tregs) [213–215]. In the context of MASH, elevated expression of ICOS and ICOSL has been correlated with disease severity, driving the expansion of pro-inflammatory Th17 cells and cytotoxic CD8⁺ T cells, which collectively exacerbate hepatic inflammation and fibrogenesis [98, 216, 217]. Notably, recent studies have reported high ICOSL expression on TREM2⁺ macrophages, a distinct subset enriched in MASH livers whose role in tissue remodelling and inflammation remains controversial, with evidence supporting both pro-fibrotic and reparative functions [98, 218, 219]. However, the expression of ICOSL on these macrophages seems to facilitate a bidirectional interaction with ICOS⁺ T cells, reinforcing pro-inflammatory circuits and promoting fibrogenesis [98, 217]. Therapeutic blockade of the ICOS–ICOSL pathway in preclinical models has demonstrated reductions in both liver inflammation and fibrosis, underscoring its potential as a promising immunomodulatory target for MASH [98, 217].

#### CD40/CD40L Pathway

CD40 is expressed on multiple hepatic cell types, including APCs, endothelial cells, and HSCs, whereas its ligand CD40L (CD154) is predominantly found on activated CD4⁺ T cells and platelets [220, 221]. The CD40–CD40L interaction promotes APC maturation and enhances the production of pro-inflammatory cytokines, serving as a key regulator of immune responses [222]. In MASH, elevated expression of both CD40 and CD40L is correlated with increased hepatic inflammation and fibrosis [167]. Activation of this pathway stimulates HSCs to synthesize ECM components, contributing directly to fibrogenesis [223]. Concurrently, CD40–CD40L signalling in macrophages promotes the secretion of pro-inflammatory cytokines such as TNF-α, IL-1β, and IL-6, further exacerbating hepatic inflammation [224]. This pathway also facilitates immune cell recruitment and bridges innate and adaptive immunity, reinforcing the chronic inflammatory environment characteristic of MASH [225]. Notably, experimental overexpression of CD40L induces severe liver injury and fulminant hepatic failure (FHF), supporting its potential as a therapeutic target in immune-mediated liver diseases [226]. However, the role of this dyad is complex. CD40 deficiency in mice exposed to obesogenic diets results in improved hepatic steatosis and metabolic parameters [227, 228]. Conversely, selective CD40 deletion in CD11c⁺ cells aggravated hepatic inflammation in MASH models, implying a context-dependent regulatory role [201]. Collectively, these conflicting findings underscore the dualistic nature of CD40/CD40L signalling in liver pathology. Further investigation is warranted to delineate its precise contribution to MASH progression and to determine whether this pathway can be safely and effectively targeted in therapeutic strategies.

#### OX40/OX40L Axis

OX40 (CD134), a member of the TNF receptor superfamily, is expressed primarily on activated CD4⁺ and CD8⁺ T cells, while its ligand OX40L is found on APCs and endothelial cells. The OX40–OX40L axis plays a crucial role in sustaining T-cell activation, proliferation, and survival, thereby amplifying adaptive immune responses [229]. In MASH, increased expression of both OX40 and OX40L has been associated with greater disease severity and heightened hepatic inflammation [196]. Engagement of this costimulatory pathway promotes the expansion of pro-inflammatory Th1 and Th17 cells, which secrete cytokines such as IFN-γ and IL-17, thereby exacerbating hepatocellular injury. Additionally, OX40 signalling impairs the function of Tregs, diminishing their immunosuppressive activity and perpetuating chronic inflammation. Experimental blockade of the OX40–OX40L interaction has been shown to attenuate liver inflammation in preclinical models, highlighting its potential as a therapeutic target in MASH [196].

### Emerging Factors Shaping Inflammation in MASLD: The Immunometabolic Perspective

**Fig. 5:**
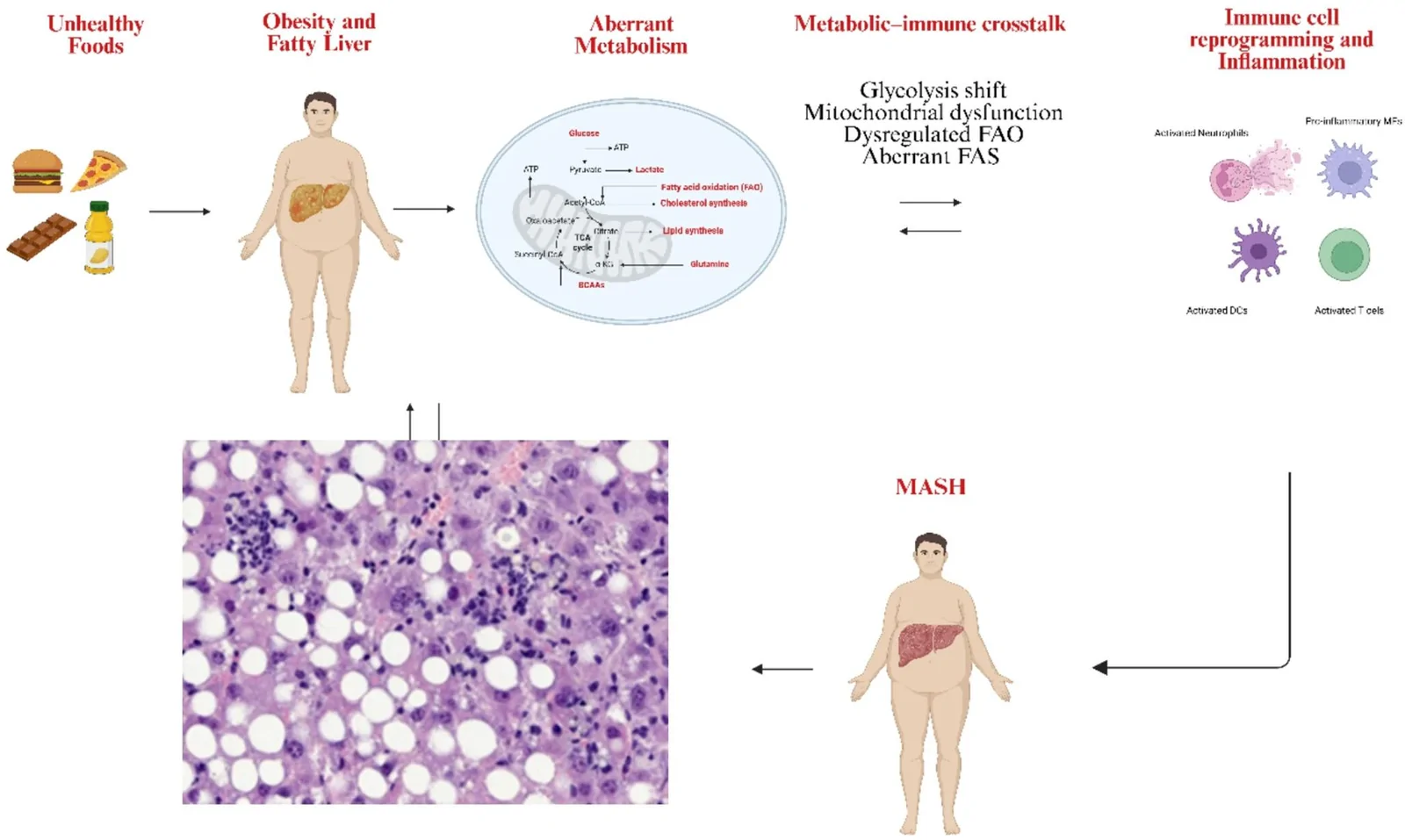
Immunometabolic crosstalk in MASH. This figure provides a conceptual schematic illustrating the bidirectional interplay between metabolic dysfunction and immune cell reprogramming during MASH progression. Diets rich in ultra-processed, calorie-dense foods promote hepatic lipid accumulation and mitochondrial dysfunction, leading to metabolic stress and altered metabolite fluxes (e.g., lactate, lipids, and branched-chain amino acids), which in turn drive immune cell metabolic rewiring and pro-inflammatory activation. Reciprocally, activated immune cells sustain hepatic metabolic disturbances through cytokine production and inflammatory signaling, establishing a self-amplifying immunometabolic loop that underlies the transition from MASLD to MASH. The histological image represents experimental evidence, showing hematoxylin and eosin (H&E) staining of liver sections from mice fed a high-fat, high-carbohydrate diet. Abbreviations: branched-chain amino acids (BCAAs); fatty acid oxidation (FAO); fatty acid synthesis (FAS). Image created with BioRender.com

Over the past decade, the emerging field of immunometabolism has gained increasing attention for its potential to explain the immune remodelling that accompanies the progression from MASLD to MASH (Fig. 5) [230]. The concept of immunometabolism posits that cellular metabolic pathways profoundly influence immune cell activation, differentiation, and effector functions [231]. This framework is particularly relevant to the pathophysiology of MASLD/MASH, as most cases develop in dysmetabolic individuals whose hepatic microenvironment is chronically exposed to macronutrient overload [232]. In this context, immune cells continuously sense metabolic and environmental cues, reprogramming their metabolism in response to them, a process that perpetuates metabolic stress, inflammation, and immune dysregulation. Importantly, these metabolic adaptations are tightly interconnected with classical inflammatory signalling pathways, including NF-κB activation, inflammasome assembly, and cytokine production, thereby amplifying and sustaining hepatic inflammation. Excess macronutrients are converted into a variety of intermediates that function not only as bioenergetic substrates but also as metabolic signals driving the reprogramming of both tissue-resident and infiltrating immune cells [233]. In line with this, accumulating evidence indicates that distinct immune cell subsets and activation states depend on specific metabolic pathways to sustain and fine-tune their effector programs. Among these factors, glycolysis has emerged as a central metabolic driver of chronic hepatic inflammation in MASLD [234, 235]. In MASLD/MASH, mitochondrial dysfunction compromises oxidative phosphorylation (OXPHOS), forcing immune cells to rely increasingly on glycolysis not only for energy production but also to sustain their pro-inflammatory activation [235]. This metabolic shift directly supports the activation of key inflammatory pathways, including NF-κB signalling and inflammasome activation, thereby linking glycolytic rewiring to cytokine production and inflammatory amplification. Enhanced glycolytic flux promotes the acquisition of an inflammatory phenotype across several innate immune cell subsets, including DCs, macrophages, and neutrophils [236]. In neutrophils, glycolysis represents the primary source of energy required to fuel effector functions such as the formation of neutrophil extracellular traps (NETs). Notably, pharmacological inhibition of key glycolytic enzymes, including hexokinase (HK) and lactate dehydrogenase A (LDHA), markedly reduces NET release [237, 238]. Similarly, macrophage activation by DAMPs upregulates glucose transporter 1 (GLUT1) expression and enhances glycolytic flux, leading to lactate accumulation. In addition to serving as a metabolic byproduct, lactate functions as a signalling molecule and an epigenetic regulator, modulating gene transcription through histone lysine lactylation [239]. This epigenetic modification promotes the expression of NOD-like receptor protein 3 (NLRP3), a key component of the inflammasome complex, thereby facilitating caspase-1 activation and the maturation of the pro-inflammatory cytokines IL-1β and IL-18, two central mediators of hepatic inflammation [235]. In parallel, DCs undergo comparable metabolic reprogramming. While quiescent DCs primarily rely on oxidative phosphorylation (OXPHOS) to sustain their basal functions, activation triggers a shift toward aerobic glycolysis, a transition that fuels their maturation, antigen presentation, and migration to lymph nodes [240]. Importantly, metabolic reprogramming in MASLD/MASH also extends to the adaptive immune compartment. Glycolysis drives the differentiation of Th1 and Th17 subsets through the induction of pyruvate kinase M2 (PKM2), which acts as a transcriptional coactivator by interacting with HIF-1α and STAT3. Pharmacological inhibition of PKM2 has been shown to suppress Th1 and Th17 polarization, thereby attenuating inflammation and disease severity in experimental models of MASLD [241, 242].

In addition to glycolysis, fatty acid oxidation (FAO) represents a central metabolic pathway that can support either pro-inflammatory or anti-inflammatory programs depending on the immune cell type and context [243]. These metabolic programs intersect with classical inflammatory signalling pathways, modulating TLR-driven responses, cytokine production, and immune cell polarization. In natural killer (NK) cells, FAO provides the energy required for cytotoxic activity and cytokine production, thereby sustaining inflammatory responses [244]. However, in MASLD/MASH, excessive lipid accumulation can dysregulate FAO, leading to mitochondrial dysfunction and impaired NK-cell effector functions [245, 246]. In dendritic cells (DCs), the outcome of fatty acid metabolism depends on the lipid species involved. Saturated fatty acids activate Toll-like receptor (TLR) pathways, promoting the expression of costimulatory molecules, MHC complexes, and pro-inflammatory cytokines. Conversely, polyunsaturated fatty acids (PUFAs) attenuate LPS-driven TLR signalling, thereby restraining DC activation. Moreover, excessive lipid loading and altered FAO may drive DCs toward a tolerogenic phenotype with reduced antigen-presenting capacity [247–249]. Macrophage polarization is similarly influenced by FAO activity. The engagement of FAO supports anti-inflammatory (M2-like) programs that promote tissue repair but may also contribute to an immunosuppressive and pro-fibrogenic microenvironment [250]. These macrophages typically express arginase-1, an enzyme that metabolizes arginine, an essential substrate for T-cell activation, thus dampening adaptive immune responses [251]. Within the adaptive lymphocyte compartment, FAO plays a pivotal role. IL-15 signalling enhances FAO in CD8⁺ T cells, sustaining their survival and effector capacity, whereas exposure to fatty acids such as linoleic acid induces a memory-like and less exhausted phenotype [252, 253]. Similarly, B-cell proliferation within germinal centers depends on FAO, highlighting the relevance of this pathway across immune compartments [254].

In parallel, fatty acid synthesis (FAS) has emerged as another essential metabolic process that shapes immune cell function. FAS deficiency impairs macrophage activity, compromising their capacity to sustain chronic inflammation [255]. Upon activation, macrophages enhance lipid synthesis via the transcription factor SREBP1-α, which supports cell proliferation and pro-inflammatory responses [179]. Notably, SREBP1-α can also promote inflammation resolution by inducing the synthesis of anti-inflammatory mediators [256]. Collectively, FAO and FAS represent versatile metabolic hubs that orchestrate immune cell fate and function. In the context of MASLD/MASH, dysregulated FAO, excessive lipid accumulation, and aberrant FAS activity converge to drive maladaptive immune responses that fuel hepatic inflammation and fibrogenesis.

Similarly, amino acid metabolism serves as a pivotal regulator of immune cell function and homeostasis in both MASLD and MASH. Perturbations in pathways controlling glutamine, arginine, and branched-chain amino acid (BCAA) turnover profoundly influence the activation, differentiation, and effector responses of both innate and adaptive immune cells [182]. In macrophages and Kupffer cells, glutaminolysis, driven by enzymes such as glutaminase (GLS) and glutamate dehydrogenase (GDH), modulates the balance between pro-inflammatory and anti-inflammatory phenotypes through mTORC1- and HIF-1α-dependent signalling. Notably, glutamine-derived α-ketoglutarate (α-KG) promotes oxidative phosphorylation (OXPHOS) and fatty acid oxidation (FAO), thereby restraining HIF-1α/NF-κB-mediated inflammatory responses, highlighting the tight coupling between amino acid metabolism and canonical inflammatory signalling pathways [257]. Similarly, dysregulated arginine metabolism, governed by the reciprocal activities of arginase-1 (ARG1) and inducible nitric oxide synthase (iNOS), critically influences nitric oxide production, macrophage polarization, and T-cell proliferation. In the adaptive compartment, amino acid availability is sensed through key metabolic sensors such as mTOR and general control nonderepressible 2 (GCN2) kinase, which dictate T-cell fate decisions by promoting effector or regulatory phenotypes. Moreover, altered tryptophan catabolism via the indoleamine 2,3-dioxygenase (IDO) pathway fosters T-cell exhaustion and immunosuppression, further exacerbating immune dysfunction in steatohepatitis [258]. In the context of hepatic metabolic stress, MASLD/MASH is characterized by systemic and intrahepatic alterations in amino acid pools, namely, decreased glutamate, serine, and glycine, alongside increased proline, hydroxyproline, and tyrosine, highlighting the strong associations among amino acid imbalance, oxidative stress, and immune dysregulation [182]. Overall, these metabolic pathways converge on key inflammatory nodes, including NF-κB, mTOR, and inflammasome activation, reinforcing the link between metabolic rewiring and immune-driven liver injury. Collectively, these metabolic derangements create an immunometabolic milieu that perpetuates hepatic inflammation, oxidative injury, and fibrogenesis, thereby driving the transition from MASLD to MASH [259]. Together with alterations in glycolysis and lipid metabolism, amino acid metabolism emerges as a fundamental axis in shaping immune responses through its integration with classical inflammatory pathways, thereby driving disease progression in MASLD/MASH (Fig. 5).

### MASH: State-of-the-art Therapies

Currently, treatment options for MASH primarily rely on lifestyle interventions, with emerging pharmacological therapies showing increasing promise. Weight loss through dietary modification and regular physical activity remains the cornerstone of MASH management, with substantial evidence supporting its efficacy in reducing hepatic inflammation and fibrosis [18, 260–262]. In this context, a cholesterol-free ketogenic diet has recently been proposed as an adjunctive strategy to increase weight loss, restore physiological glucose metabolism, and mitigate hepatic inflammation and fibrosis in a murine model of MASH [263]. These benefits are further supported by independent studies demonstrating the protective role of hepatic ketogenesis in metabolic liver disease [264, 265], although the overall efficacy of such dietary interventions remains debated. Regarding pharmacotherapy, resmetirom, a selective thyroid hormone receptor beta (THR-β) agonist, was recently approved by the FDA for the treatment of non-cirrhotic MASH patients with moderate to advanced liver fibrosis [266]. In addition, glucagon-like peptide-1 receptor agonists (GLP-1 RAs), such as semaglutide, which is commonly used in type 2 diabetes and obesity management, are being evaluated for their beneficial effects on liver histology in MASH [267]. Overall, effective MASH management requires a multidisciplinary approach that addresses not only liver pathology but also associated metabolic comorbidities such as type 2 diabetes, dyslipidemia, and hypertension. Nonetheless, the absence of approved therapies for advanced stages of MASH, including cirrhosis, highlights an urgent need for continued research and therapeutic development.

### Future Perspectives

To date, most therapeutic strategies for MASH have focused on correcting underlying metabolic imbalances [268]. While this approach is beneficial in obese or metabolically dysregulated individuals, either to prevent disease onset or slow progression, its long-term efficacy, particularly in advanced stages, remains uncertain [268]. This limitation is especially relevant given that most cases are diagnosed late, when cirrhosis or HCC has already developed [269].

Chronic inflammation has emerged as a key driver of fibrogenesis and malignant transformation, fueled by oxidative stress, sustained tissue injury, hypoxia, and immune exhaustion resulting from persistent immune activation [270–272]. Increasing evidence suggests that inflammatory and immunological mechanisms are not merely secondary to metabolic dysfunction, but represent central determinants of disease progression, supporting the development of targeted therapeutic strategies.

Accordingly, multiple pathways have been identified as potential therapeutic targets with varying degrees of translational maturity. At the clinical and late preclinical stages, strategies aimed at blocking monocyte recruitment (e.g., CCR2/CCR5 axis) and modulating cytokine signaling (e.g., IL-1β, TNF-α) have shown promise in reducing hepatic inflammation and fibrosis, although their long-term efficacy and safety remain under investigation [273–276]. Notably, the dual CCR2/CCR5 antagonist cenicriviroc failed to meet its primary endpoint in the phase III AURORA trial, underscoring the challenges in translating preclinical findings into clinical benefit [274, 277].

Similarly, cytokine-targeting approaches are being explored. IL-17-driven responses have been implicated in hepatic inflammation and fibrogenesis, suggesting that modulation of this pathway may hold therapeutic potential [278]. However, current clinical data remain limited and partly controversial, with available studies indicating an acceptable safety profile but lacking robust evidence of efficacy, thus requiring validation in larger cohorts [279, 280].

Immune checkpoint pathways have also been implicated in shaping hepatic immune responses and T-cell dysfunction in MASH, although their therapeutic targeting remains largely exploratory and appears less effective in MASH-related HCC compared to tumors of viral etiology [209, 210].

Importantly, these approaches highlight key challenges in immunomodulation. Immune pathways in MASLD/MASH often exert context-dependent roles, with the coexistence of pathogenic and restorative immune subsets contributing both to tissue injury and repair. As a result, broad immunosuppression may disrupt beneficial immune populations, including restorative macrophages, ultimately limiting therapeutic efficacy [27]. Moreover, excessive immune suppression may impair host defense, increase susceptibility to infections, and potentially promote tumor immune escape, thereby favoring progression toward hepatocellular carcinoma (HCC) [281].

Similarly, targeting fibrogenic pathways such as TGF-β signaling or hepatic stellate cell activation represents a well-established antifibrotic approach currently under active development [282].

In parallel, emerging strategies are exploring the modulation of adaptive immunity and costimulatory pathways, including the ICOS/ICOSL, CD40/CD40L, and OX40/OX40L axes [98, 201, 283]. Although still largely preclinical, these approaches may disrupt the self-reinforcing interplay between innate and adaptive immunity that sustains chronic liver inflammation [284].

Another promising frontier is immunometabolism. Targeting key metabolic checkpoints, such as glycolysis (e.g., PKM2), fatty acid oxidation, and amino acid sensing pathways (e.g., mTOR signaling) [285–287], may enable selective reprogramming of pathogenic immune responses, offering the possibility of simultaneously addressing metabolic dysfunction and inflammation.

Further investigation of the crosstalk between immune cells and fibrogenic populations, particularly T lymphocytes and myofibroblasts, represents an additional avenue for therapeutic innovation, as these interactions are central to progression toward fibrosis, cirrhosis, and HCC. In cases where HCC has already developed, therapeutic strategies should also aim to reinvigorate anti-tumor immunity, for example through immune checkpoint modulation or reversal of T-cell exhaustion, thereby linking MASH pathogenesis to cancer immunotherapy.

Beyond the mechanisms discussed above, several emerging concepts are likely to shape future research in MASLD and MASH. Increasing evidence suggests that environmental exposures may act as important modifiers of disease susceptibility and progression. In addition to classical endocrine-disrupting chemicals, growing attention is being devoted to heavy metals and other environmental pollutants that can promote oxidative stress, mitochondrial dysfunction, immune activation, and epigenetic alterations. These observations support the concept of MASLD as an exposome-influenced disease, resulting from the interaction between genetic predisposition, metabolic dysfunction, and lifelong environmental exposures [22].

Another rapidly evolving area concerns the interplay between immunometabolism and epigenetic regulation. Metabolic stress not only alters immune cell function but may also induce persistent epigenetic remodeling through changes in DNA methylation, chromatin accessibility, histone modifications, and non-coding RNA expression. Such mechanisms may contribute to trained immunity, a form of innate immune memory that sustains inflammatory responses even after removal of the initial trigger. Understanding how immunometabolic signals shape long-term inflammatory programs may provide novel opportunities for therapeutic intervention [288–290].

Recent advances in single-cell and spatial transcriptomic technologies have also revealed an unexpected degree of complexity within the hepatic macrophage compartment. The progressive replacement of resident Kupffer cells by bone marrow-derived macrophages and the emergence of specialized populations associated with fibrosis and tissue remodeling suggest that macrophage heterogeneity may represent a critical determinant of disease outcome. Future studies will be required to define the functional specialization of these populations and to identify strategies capable of selectively targeting pathogenic macrophage subsets while preserving reparative responses [291–293].

Finally, DAMP-sensing pathways are increasingly recognized as central mediators linking metabolic stress to chronic inflammation. Among these, the HMGB1–RAGE axis has emerged as a promising candidate driving sustained NF-κB activation, inflammasome signaling, immune dysfunction, and fibrogenesis. HMGB1, advanced glycation end products (AGEs), and members of the S100 protein family have all been implicated as relevant RAGE ligands in chronic liver injury. A better understanding of how these pathways integrate metabolic, inflammatory, and epigenetic signals may facilitate the development of more effective and personalized therapeutic approaches. Collectively, these emerging mechanisms support a shift from a purely metabolic view of MASLD toward a broader framework integrating environmental exposures, immune remodeling, and epigenetic regulation. Such an approach may help explain disease heterogeneity and accelerate the development of precision medicine strategies for patients with MASLD and MASH [294–296].

All together, these considerations suggest that effective treatment of MASLD/MASH will likely require stage-specific and combinatorial therapeutic strategies, simultaneously targeting metabolic dysfunction, inflammation, and fibrogenesis.

## Conclusion

In conclusion, chronic inflammation emerges as a central driver in the pathogenesis and progression of MASLD and MASH, orchestrating a complex interplay between metabolic dysfunction and immune activation. Innate immune cells, including Kupffer cells, monocytes, neutrophils, and NKT cells, continuously sense metabolic and lipotoxic stress, sustaining inflammatory signaling that primes adaptive immune responses. T and B lymphocytes further amplify this inflammatory milieu, with costimulatory molecules critically shaping lymphocyte activation, differentiation, and persistence within the inflamed liver microenvironment. Beyond immune cells, both parenchymal hepatocytes and non-parenchymal cell types such as HSCs, liver sinusoidal endothelial cells, and cholangiocytes actively contribute to perpetuating inflammation, tissue remodeling, and fibrogenesis. Within this complex network, cytokines, chemokines, and hepatokines act as key mediators of intercellular communication, linking immune activation to metabolic dysfunction. Importantly, immunometabolism, situated at the crossroads of nutrient sensing, mitochondrial activity, and inflammatory signaling, emerges as a pivotal mechanism integrating metabolic stress with chronic immune activation.

Collectively, these insights underscore the concept that MASLD/MASH is a systemic immunometabolic and inflammatory disease, rather than a condition driven solely by metabolic dysfunction. This perspective is highly relevant to the field, as it highlights emerging mechanistic pathways that may serve as promising targets for therapeutic intervention. By elucidating how chronic inflammation and immunometabolic processes shape disease progression, this review article provides a framework for developing more precise and effective strategies aimed at interrupting inflammatory circuits, modulating immune responses, and ultimately restoring hepatic homeostasis.

## Data Availability

No datasets were generated or analysed during the current study.
